# Effects and Safety of Oral Iron for Heart Failure with Iron Deficiency: A Systematic Review and Meta-Analysis with Trial Sequential Analysis

**DOI:** 10.1155/2022/6442122

**Published:** 2022-09-17

**Authors:** Nannan Tan, Yiqing Cai, Junjie Liu, Xiaoping Wang, Lin Ma, Guanjing Ling, Jinchi Jiang, Qiyan Wang, Yong Wang

**Affiliations:** ^1^School of Traditional Chinese Medicine, Beijing University of Chinese Medicine, Beijing, China; ^2^Beijing Key Laboratory of TCM Syndrome and Formula, Beijing, China; ^3^Key Laboratory of Beijing University of Chinese Medicine, Ministry of Education, China; ^4^Department of Cardiology, Nanjing Pukou Hospital of Traditional Chinese Medicine, Nanjing, China; ^5^School of Life Science, Beijing University of Chinese Medicine, Beijing, China

## Abstract

**Background:**

Oral iron supplement is commonly prescribed to heart failure patients with iron deficiency. However, the effects of oral iron for heart failure remain controversial. This study included randomized controlled trials (RCTs) for meta-analysis to evaluate the effects of oral iron for heart failure patients.

**Methods:**

Nine databases (The Cochrane Library, Embase, PubMed, CINAHL, Web of science, CNKI, SinoMed, VIP, and Wanfang) were searched for RCTs of oral iron for heart failure from inception to October 2021. The effects were assessed with a meta-analysis using Revman 5.3 software. The trial sequential analysis was performed by TSA 0.9.5.10 beta software. The risk of bias of trials was evaluated via Risk of Bias tool. The evidence quality was assessed through GRADE tool.

**Results:**

Four studies including 582 patients with heart failure and iron deficiency were enrolled. The results indicated that oral iron treatment could improve left ventricular ejection fraction (LVEF, MD = 1.52%, 95% CI: 0.69 to 2.36, *P* = 0.0003) and serum ferritin (MD = 1.64, 95% CI: 0.26 to 3.02, *P* = 0.02). However, there was no between-group difference in the 6-minute walk distances (6MWT), N terminal pro B type natriuretic peptide (NT-proBNP) or hemoglobin level when compared with control group. Subgroup analyses revealed that the effects of oral iron on 6 MWT and serum ferritin could not be affected by duration and frequency of oral iron uptakes. In trial sequential analysis of LVEF and serum ferritin, the Z-curves crossed the traditional boundary and trail sequential monitoring boundary but did not reach the required information size.

**Conclusion:**

This analysis showed that oral iron could improve cardiac function measured by LVEF, and iron stores measured serum ferritin, but lack of effect on exercise capacity measured by 6 MWT, and iron stores measured by hemoglobin. Given the overall poor methodological quality and evidence quality, these findings should be treated cautiously.

## 1. Introduction

Heart failure (HF) is the end stage of a variety of heart diseases that affects approximately 40 million people around the world [1]. It has been reported that the incidence of HF in Europe is about 3/1000 person-years (all age-groups) and the overall incidence is increasing due to the increased aging population [2, 3]. In China, the prevalence of HF among adults aged over 35 is 1.3%, and the mortality rate of inpatients with HF is 4.1% [4, 5]. In the USA, 6 million people are afflicted with HF, and it is estimated that there will be over 8 million people with HF by 2030 [6, 7].

Some studies showed that iron deficiency was a risk factor for HF patients, and approximately 50% of patients with HF had low levels of available iron [8–10]. The pathogenesis of iron deficiency occurring in heart failure is not clear. Gastrointestinal blood loss, renal failure, and inflammation may be involved [11]. And transferrin and hepcidin are essential serum proteins related to iron metabolism. Transferrin is mainly responsible for the delivery of iron via transferrin-receptor that is a potential biomarker to identify iron deficiency in HF patients [12]. Moreover, high level hepcidin, which is a vital regulator of systemic iron metabolism, can block iron absorption, ultimately leading to iron deficiency [13, 14]. Iron deficiency can cause increased cardiac output, left ventricular hypertrophy, and left ventricular dilation, leading to symptomatic chronic heart failure [11]. Just as Naito et al. indicated that iron-deficient diet could induce anemia, which would eventually lead to left ventricular hypertrophy [15]. Hence, the effects of iron deficiency in HF have gained increased attention in recent years, and iron supplementation is considered as an attractive treatment strategy for HF [16]. Intravenous iron has been becoming prevalent in recent years due to the less toxicity and high efficiency. Ferric carboxymaltose, iron sucrose, iron isomaltoside, sodium ferric gluconate, etc. are common intravenous iron preparations [17]. Several studies indicated that intravenous iron supplementation could improve symptoms, quality of life and length of hospital stay in patients with HF [18–22]. The American College of Cardiology's 2017 Guidelines for the Prevention of HF demonstrated that the symptoms of HF patients with iron deficiency could be improved by intravenous iron injection [23]. In addition, intravenous iron supplementation with ferric carboxymaltose was recommended for patients with HF in 2021 European Society of Cardiology (ESC) Guidelines and the level of evidence was A [24]. Pezel et al. found that about 39.3% HF patients with iron deficiency received intravenous iron supplementation in French [25]. However, hypophosphatemia and injection reactions at the injection site were observed when patients received intravenous [26, 27].

In addition to intravenous iron injection, oral iron is frequently prescribed to patients with iron deficiency [28]. Ferrous sulfate, ferrous gluconate, and ferrous fumarate are common preparations in clinical [29]. The HF patients received oral iron supplementation even more frequent than intravenous supplementation [30]. However, it is not recommended in HF guidelines. There may be several reasons. Firstly, oral iron supplementation is not as effective as intravenous iron supplementation in HF patients with iron deficiency [31]. Secondly, intestinal functions are usually compromised in patients with HF due to inadequate oxygen supply and this will affect iron absorption [32]. Lewis et al. suggested that oral iron supplementation could not significantly improve exercise ability in HF patients with reduced ejection fraction and is therefore not useful for HF patients [33]. However, other studies suggested that oral iron may be beneficial for HF patients [34, 35]. The results of randomized controlled trials (RCTs) were inconsistent and the evidence was inadequate [36]. Hence, the efficiency of oral iron in the treatment of HF awaits further investigations [37].

In this study, we performed a systematic review and meta-analysis of RCTs to assess the effects and safety of oral iron in the treatment of HF patients with iron deficiency. In addition, we explored whether the effects were influenced by the frequency and duration of oral iron uptakes.

## 2. Methods

### 2.1. Registration

The protocol of this systematic review and meta-analysis was registered on PROSPERO under the number CRD42021282982.

### 2.2. Search Strategy

**Figure 1:**
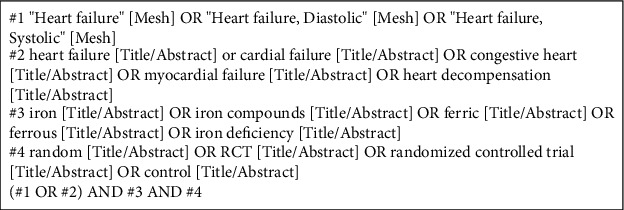
The search strategy of PubMed.

The Cochrane Library, Embase, PubMed, the Cumulative Index to Nursing and Allied Health Literature (CINAHL), Web of Science, China National Knowledge Infrastructure (CNKI), Chinese Biomedical Literature Service System (SinoMed), Chinese Scientific Journals (VIP) Database, and Wanfang Data Chinese database (Wanfang) were searched by two authors independently from inception to October 2021. English search terms included: heart failure, cardiac failure, congestive heart, myocardial failure, heart decompensation, iron, iron compounds, ferric, ferrous, and iron deficiency. Chinese search terms included: xin_li_shuai_jie, xin_shuai, tie, ya_tie, er_jia_tie, san_jia_tie, tie_ji, and tie_que_fa. Only RCTs published in English or Chinese language were included. Taking PubMed as an example, specific search strategies were shown in Figure 1.

### 2.3. Inclusion and Exclusion Criteria

According to 2021 ESC Guidelines for the diagnosis and treatment of acute and chronic HF [24], RCTs that enrolled HF patients (no type of restriction) with ejection fraction threshold <50% and serum ferritin lower than 100 ng/mL or serum ferritin 100-299 ng/mL with transferrin saturation (TSAT) <20% were included. The intervention included oral iron only, regardless of treatment duration. The comparisons included usual care, placebo, or other comparators, regardless of treatment duration. The primary outcomes were left ventricular ejection fraction (LVEF), 6-minute walk distances (6 MWT), and serum ferritin. The secondary outcomes were N terminal pro B type natriuretic peptide (NT-proBNP), hemoglobin, the quality of life, safety, and adverse events.

Studies with the following conditions were excluded: (1) duplicate articles and (2) articles where data reports were incomplete, or data were not available.

### 2.4. Study Selection and Data Extraction

After deleting duplicate literature, two reviewers screened the titles and abstracts independently by using Endnote X9 software. Studies relevant to the purpose of this review were included to read the full texts. Reference lists of relevant studies were also reviewed to supplement the missing studies. Discrepancies were resolved by a third reviewer. The selection procedure was shown in a Preferred Reporting Items for Systematic Reviews and Meta-Analyses (PRISMA) flowchart.

The following information from the included studies was extracted: first author, year of publication, the diagnosis of HF and iron deficiency, sample size, characteristic of participants (age, gender), intervention, treatment duration, and outcomes. Data extraction was performed by two reviewers independently.

### 2.5. Quality Assessment

#### 2.5.1. Risk of Bias

The assessment of risk of bias for RCTs was performed independently by two authors using the Risk of Bias (RoB) tool mentioned in Cochrane Handbook for Systematic Reviews of Interventions [38]. Selection bias (random sequence generation and allocation concealment), performance bias (blinding of participants and personnel), detection bias (blinding of outcome assessors), attrition bias, reporting bias, and other sources of bias were assessed. In accordance with the RoB, for each bias judgement could be “low risk”, “high risk”, or “unclear risk”.

#### 2.5.2. Quality of Evidence

The quality of evidence was estimated by the standard Grading of Recommendation Assessment, Development and Evaluation (GRADE) tool. Five domains were assessed by two researchers, including (1) risk of bias; (2) inconsistency in the results; (3) indirectness of evidence; (4) imprecision of evidence; (5) publication bias. One domain with a serious problem can degrade the quality by one level, when there is a very serious problem with the domain, this can reduce the quality by two levels. The quality of evidence for outcomes could be rated into four levels: very low, low, moderate, or high using this standard approach [39].

### 2.6. Statistical Analysis

Data analysis was performed using RevMan 5.3 software. Continuous variables were presented as mean difference (MD) or standard mean difference (SMD), with 95% confidence intervals (CI). *P* < 0.05 was defined as statistically significant. The *I*^2^ test and *χ*^2^ test were conducted to quantify the statistical heterogeneity between trials. A fixed-effects model was used when *P* ≥ 0.1 and *I*^2^ < 50% and the PICOs of the trials in the meta-analysis had no obvious clinical diversity, while a random-effects model was used when *P* < 0.1 or *I*^2^ ≥ 50%. The one-study-omission sensitivity analysis was conducted to determine the possible source of heterogeneity. Subgroup analyses based on treatment durations and frequency of oral iron were performed to explore the possible dose response relationship between the intervention characteristics and changes in outcomes when sufficient trials were included. The trial sequential analysis (TSA) was performed by TSA 0.9.5.10 beta software to verify the stability of the results and to estimate the total sample size required for meta-analysis. Publication bias was analyzed by funnel plot when at least 10 trials were included in a meta-analysis.

## 3. Results

### 3.1. Study Identification and Characteristic

**Figure 2:**
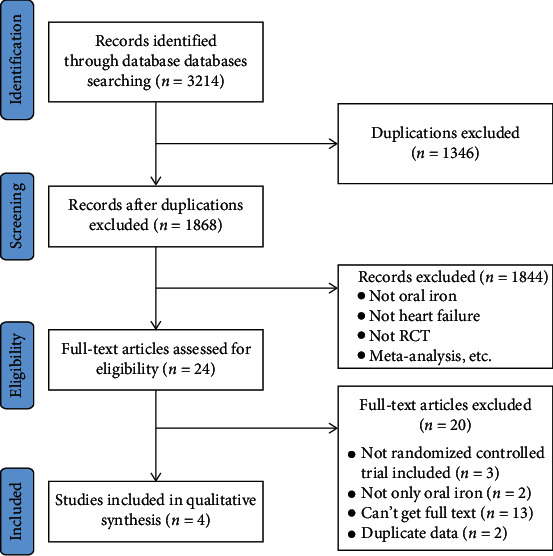
PRISMA flow diagram for selection of studies.

The original search retrieved 3214 eligible studies. 1346 duplicated studies were excluded. Further screening excluded 1844 studies based on titles and abstracts, and 24 studies were selected. Four studies [33, 40–42] were included after the full texts were reviewed. The data retrieving process was shown in Figure 2.

The four studies enrolled 582 patients, assigned to oral iron group (*n* = 290) versus control group (*n* = 292). One study was taken place in the United States, one in Serbia, and two in China. The specific information was shown in Table 1.

### 3.2. Risk of Bias Assessment

**Figure 3:**
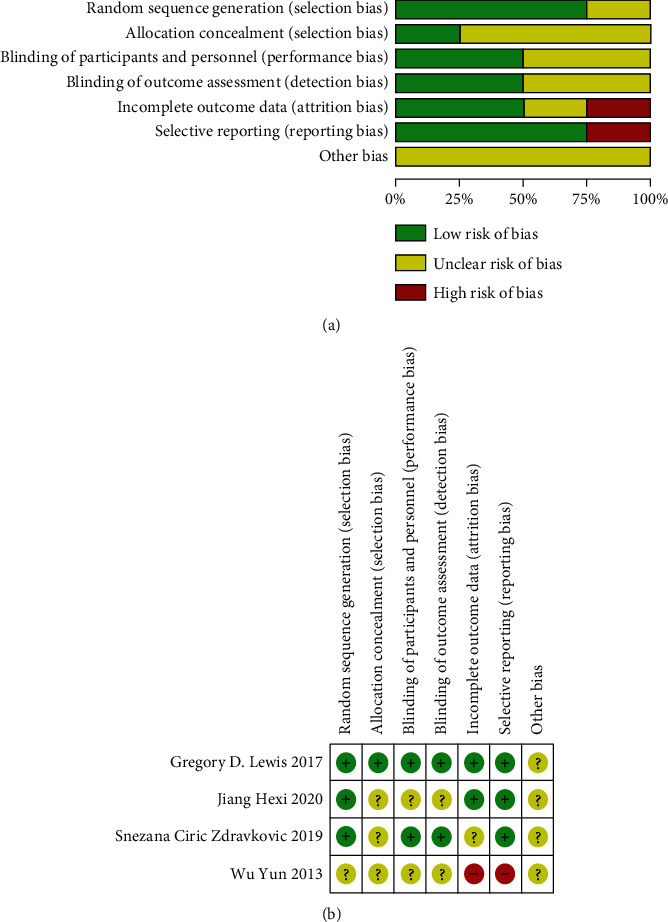
(a) Risk of bias assessment across all included studies; (b) risk of bias assessment for each study.

We identified the high overall risk of bias for all trials because at least one domain in each of these trials was judged to be at unclear or high risk of bias. As shown in Figures 3(a) and 3(b), one trial (25%) did not provide the method of random sequence generation and three trials (75%) did not report the method of allocation concealment. Therefore, they were assessed as having unclear risk of selection bias. The performance bias was rated as unclear risk because two trials (50%) did not mention the information about blinding of participants and personnel, and the detection bias was unclear risk because two trials (50%) did not report the information about blinding of outcome assessment. One trial (25%) did not provide the information about incomplete outcome data, and one trial (25%) mentioned one case of loss to follow-up, indicating that there was attrition bias. Reporting bias was detected in one trial (25%) because the results of some outcomes included were not reported.

### 3.3. Primary Outcome

#### 3.3.1. LVEF

**Figure 4:**
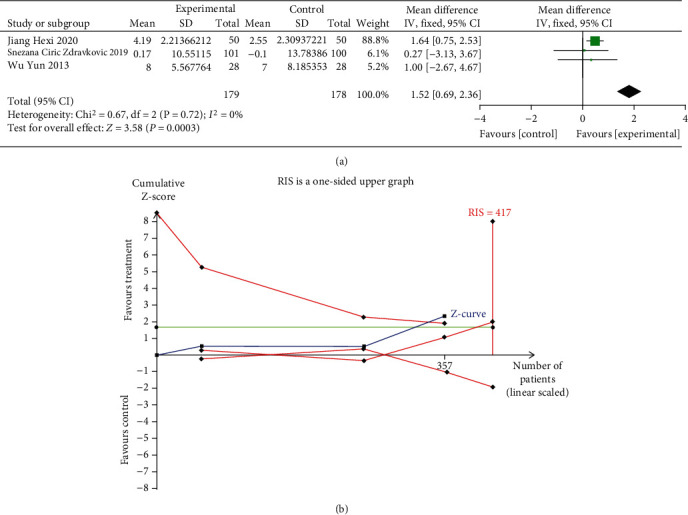
(a) Forest plots of the effect of oral iron on LVEF; (b) trial sequential analysis of LVEF.

Three RCTs (with 357 patients) reported the effects of oral iron on LVEF. The meta-analysis with a fixed-effects model showed that the LVEF was improved by oral iron administration (MD = 1.52%, 95% CI: 0.69 to 2.36, *P* = 0.0003, *I*^2^ = 0%, Figure 4(a)).

The result of the TSA on LVEF showed that the cumulative Z-curves crossed both the conventional boundary and the trial sequential monitoring boundary, indicating the reliability of the meta-analysis result that support the benefit of oral iron for HF. However, the cumulative sample size did not reach the required information size of 417. As shown in Figure 4(b).

#### 3.3.2. 6-Minute Walk Distances

**Figure 5:**
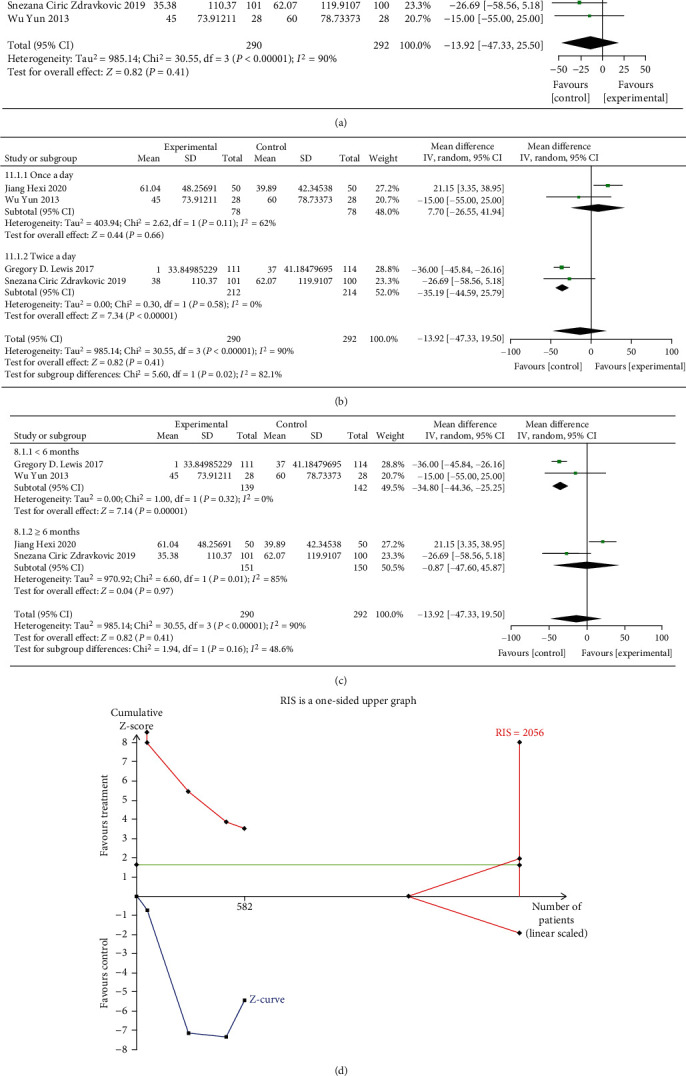
(a) Forest plots of the effect of oral iron on 6 MWT; (b) subgroup analysis of treatment frequency on 6 MWT; (c) subgroup analysis of treatment duration on 6 MWT; (d) trial sequential analysis of 6 MWT.

**Figure 6:**
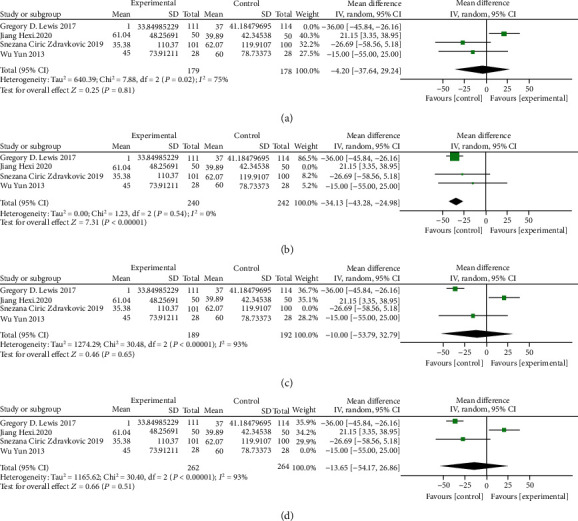
Sensitivity analysis of 6-minute walk distances.

The result of meta-analysis of 4 RCTs (582 patients) with a random-effects model illustrated that there was no significant effect of oral iron on 6 MWT (MD = −13.92 m, 95% CI: -47.33 to 19.50, *P* = 0.41, *I*^2^ = 90%, Figure 5(a)). Sensitivity analysis showed that when the study of Jiang Hexi was removed, the *I*^2^ decreased to 0%, as shown in Figure 6.

Due to the availability of the number of trials, subgroup analyses were performed on 6 MWT. The subgroup analysis based on treatment frequency showed that taking oral iron either once a day (MD = 7.7, 95% CI: -26.55 to 41.94, *P* = 0.66, *I*^2^ = 62%) or twice a day (MD = −35.19, 95% CI: -44.59 to -25.79, *P* < 0.00001, *I*^2^ = 0%) had no difference of effect on 6 MWT (Figure 5(b)). In the subgroup of treatment duration, oral iron treatment lasting less 6 months (MD = −34.80, 95% CI: -44.36 to -25.25, *P* < 0.00001, *I*^2^ = 0%) or over 6 months (MD = −13.92, 95% CI: -47.33 to 19.50, *P* = 0.97, *I*^2^ = 85%) could not improve 6 MWT in HF patients with iron deficiency (Figure 5(c)).

The result of the TSA on 6 MWT showed that the cumulative Z-curves did not crossed both the conventional boundary and the trial sequential monitoring boundary, indicating no potential advantages for oral iron on 6 MWT. However, the cumulative sample size did not reach the required information size of 2056 (Figure 5(d)).

#### 3.3.3. Serum Ferritin

**Figure 7:**
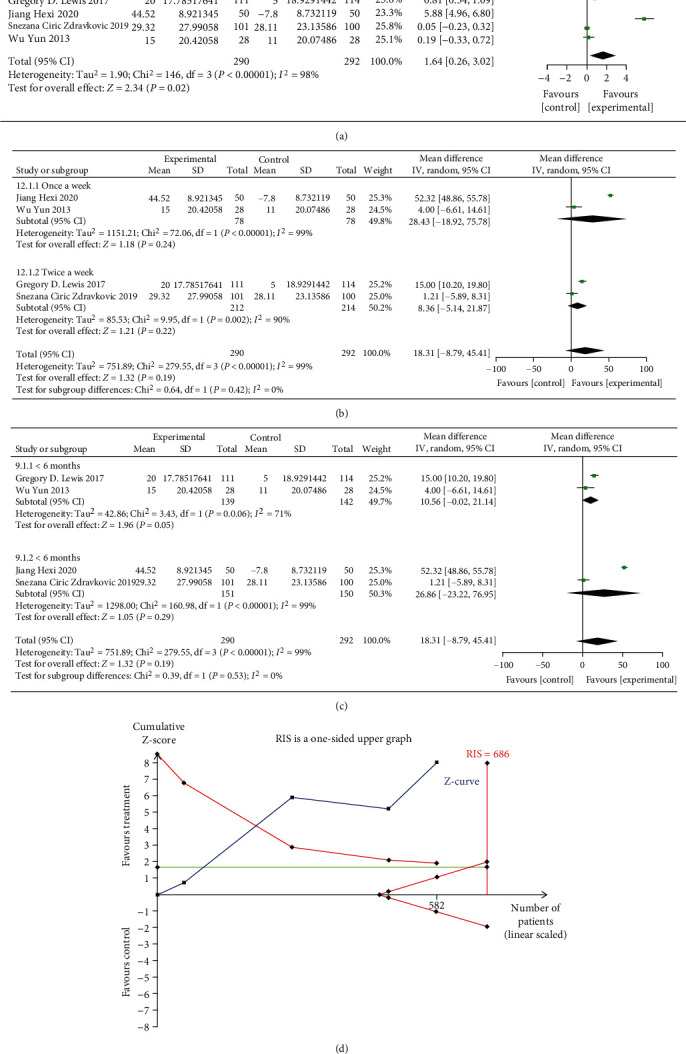
(a) Forest plots of the effect of oral iron on serum ferritin; (b) subgroup analysis of treatment frequency on serum ferritin; (c) subgroup analysis of treatment duration on serum ferritin; (d) trial sequential analysis of serum ferritin.

**Figure 8:**
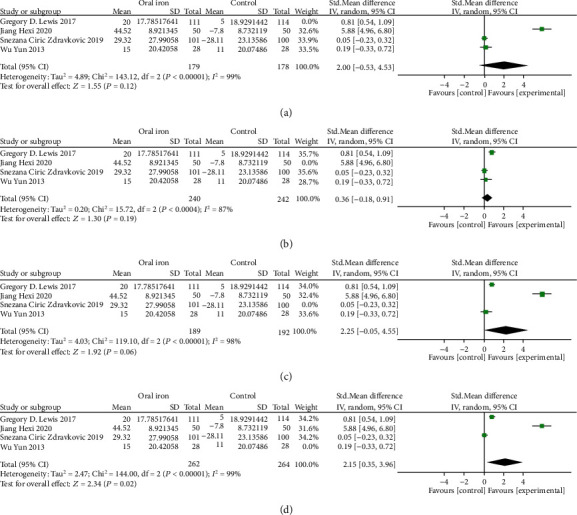
Sensitivity analysis of serum ferritin.

The result of meta-analysis of 4 RCTs (582 patients) with a random-effects model showed that oral iron could improve serum ferritin level (MD = 1.64, 95% CI: 0.26 to 3.02, *P* = 0.02, *I*^2^ = 98%, Figure 7(a)). Sensitivity analysis showed that the heterogeneity was not obviously decreased when four studies were removed one by one, as shown in Figure 8.

The subgroup analyses of treatment frequency and duration on serum ferritin illustrated that no dose-response relationships were identified between the frequency of oral iron and changes in serum ferritin (Figures 7(b) and 7(c)).

The result of the TSA on serum ferritin showed that the cumulative Z-curves crossed both the conventional boundary and the trial sequential monitoring boundary, indicating the reliability of the meta-analysis results that support the benefits of oral iron for HF patients. However, the cumulative sample size did not reach the required information size of 686. As shown in Figure 7(d).

### 3.4. Secondary Outcomes

#### 3.4.1. Hemoglobin

**Figure 9:**
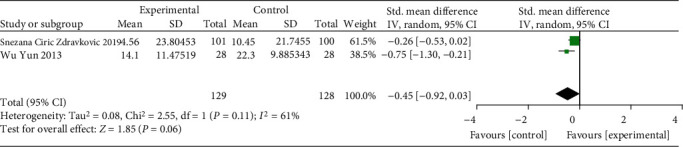
Forest plots of the effect of oral iron on hemoglobin.

The result of meta-analysis of 2 RCTs (257 patients) with a random-effects model reported that oral iron had no effect on hemoglobin (SMD = −0.45, 95% CI: -0.92 to 0.03. *P* = 0.06, *I*^2^ = 61%, Figure 9).

#### 3.4.2. NT-proBNP

**Figure 10:**
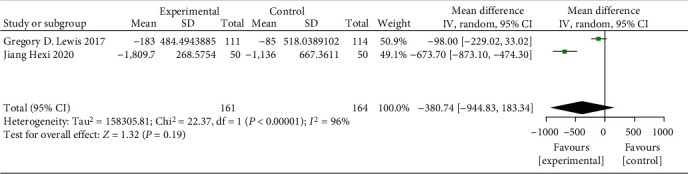
Forest plots of the effect of oral iron on NT-proBNP.

The result of meta-analysis of 2 RCTs (325 patients) with a random-effects model showed that the level of NT-proBNP was not reduced by oral iron (MD = −380.74 pg/mL, 95% CI: -994.83 to 183.34, *P* = 0.19, *I*^2^ = 96%, Figure 10).

### 3.5. Quality of Life

Two studies reported the quality of life. Lewis et al. used the Kansas City Cardiomyopathy (KCCQ) to estimate the quality of life in patients with HF. The result showed that oral iron could not improve quality of life scores at the end of 16 weeks. However, the study by Jiang Hexi revealed that oral iron was beneficial for the Minnesota Living with Heart Failure Questionnaire (MLHFQ) scores at the end of 24 weeks, not 16 weeks.

### 3.6. Safety of Oral Iron

Three studies reported adverse events. The RCT by Snezana Ciric Zdravkovic reported that patients taking ferric supplements had sporadic intolerance, but no patients stopped taking iron supplements during the experiment. The study by Jiang Hexi found that mild nausea occurred in oral iron group, but none of the patients quit the study either. Lewis et al. found that the adverse events were similar between oral iron and placebo group. Overall, the adverse events of oral iron seemed to be mild and tolerable.

### 3.7. Publication Bias

The publication bias was not analyzed because the number of RCTs reported each outcome was less than 10.

### 3.8. Quality of Evidence

The evidence quality of the effects of oral iron on LVEF and 6 MWT was low, and the evidence quality on NT-proBNP, serum ferritin, and hemoglobin were very low (Table 2).

## 4. Discussion

The main findings of this systematic review and meta-analysis indicated that oral iron could improve cardiac function measured by LVEF and iron stores measured by serum ferritin, but lack of effect on exercise capacity measured by 6 MWT and iron stores measured by hemoglobin. And there were no dose-response relationships between the frequency and duration of oral iron and changes in 6MWT and serum ferritin.

6 MWT, NT-proBNP, and hemoglobin were not improved by oral iron in our study. However, oral iron supplementation could improve LVEF and serum ferritin. TSA can compensate for the risk of random errors producing in traditional meta-analysis and estimate the required amount of information and the stability of the result of meta-analysis. Moreover, the false positive result can be prevented effectively. In our study, the results of TSA on LVEF and serum ferritin supported the benefits of oral iron for HF patients in meta-analysis, but not reached the required information size, further investigations were needed to support the stability of result. Considering the high heterogeneity of serum ferritin and the possible source was not tracked, the result of serum ferritin should be treated with caution. The sensitivity analysis of 6 MWT showed that when the study of Jiang Hexi was removed, the heterogeneity became low. The study might be the source of high heterogeneity. Tracing back to the original study, we found that the control group in this study was blank, but control groups in other studies were not. We speculated that the control group might be a factor influencing the result.

The subgroup analysis showed that no dose-response relationships were identified between the frequency and duration of oral iron and changes on 6 MWT and serum ferritin. However, some studies have shown that daily take of low dose of iron supplementation was better than high dose for treating anemia in pregnant patients, and alternate day was better than daily iron supplementation [26, 43]. The study by Moretti et al. had similar conclusion [44]. Hence, we still suggest that the effect of different dose and duration of oral iron require further research although our result is negative.

Iron is vital for numerous biological processes. The heart is more susceptible to iron deficiency because iron is a metal cofactor participating in the formation of mitochondrial enzymes that support the high energy requirements of myocardial contraction [45–47]. Fatigue, poor physical performance and decreased exercise tolerance has been observed in HF patients with iron deficiency [48]. Iron supplement, especially intravenous products, is beneficial to improve 6 MWT, peak oxygen consumption, and quality of life [9]. HF patients with iron deficiency, regardless the anemia status, intravenous iron therapy should be considered [36]. In addition to intravenous iron supplement, oral iron is often prescribed to HF patients with iron deficiency. Pezel et al. found that 40 of 168 HF patients received oral iron treatment in French [25]. And more than 90% patients received oral treatment in four Europe countries [49]. However, the effects of oral iron for HF patients with iron deficiency are still controversial. The poor absorption of oral iron may affect the effects for HF patients with iron deficiency. Firstly, drugs such as proton pump inhibitors (PPIs) and histamine H2 receptor antagonist (H2RA) which have potential effectiveness in treating HF can interfere the absorption of iron [50, 51]. Secondly, patients with HF may develop intestinal edema after venous congestion due to increased pressure in the right atrium [52]. The cardiac output, systemic circulating blood flow, and intestinal wall barrier may decrease during heart failure [32, 53]. Intestinal function should be taken into consideration when prescribing oral iron to HF patients [54]. In addition, iron absorption is reduced due to inflammation which is involved in the pathogenesis and progression of HF [28, 55]. According to the findings of this meta-analysis, although oral iron is beneficial to LVEF and serum ferritin, more evidence for oral iron preparations in HF patients is still needed.

This study had some limitations. Firstly, we only included studies of HF patients with LVEF <50% and the extensibility of conclusions is limited. Secondly, we focused mainly on surrogate endpoints and did not include key patient-oriented outcomes such as HF hospitalization, and death. Thirdly, there was a lack of high quality RCTs of oral iron on heart failure and the number of enrolled studies was relatively small. Fourthly, we could not conduct separate meta-analysis based on different comparisons, i.e., placebo or blank control, because the number of included studies is insufficient. Finally, the publication bias was not analyzed because the number of each outcome was less than 10.

## 5. Conclusion

Oral iron could improve cardiac function measured by LVEF, and iron stores measured by serum ferritin, but lack of effect on exercise capacity measured by 6 MWT, and iron stores measured by hemoglobin. Given the overall poor methodological quality and evidence quality, these findings should be treated cautiously. More high-quality RCTs are needed in the future.

## Figures and Tables

**Table 1:** Characteristic of the included trials.

Study	HF diagnosis	Iron deficiency diagnosis	Sample size	Age (year)		Gender (male/female)		Intervention		Treatment duration	Outcomes
			Treatment /control	Treatment	Control	Treatment	Control	Treatment	Control		
Jiang HX 2020 [42]	NYHA through II to III and LVEF <40%	Serum ferritin levels <100 *μ*g/L	50/50	69.12 ± 13.07	69.86 ± 14.74	31/19	33/17	Polysaccharide iron complex, 150 mg, once daily	Blank control	24 weeks	①②③⑤⑥⑦
Snezana CZ 2019 [40]	Chronic decompensated HF	Serum ferritin levels <100 *μ*g/L and transferrin saturation levels <20%	101/100	70.76 ± 9.81	73.3 ± 9.77	66/35	56/44	Oral ferric hydroxide polymaltose complex, without ascorbic acid	Oral ferrous fumarate, ascorbic acid, twice daily	24 weeks	②③④⑤⑦
Gregory DL 2017 [33]	NYHA through II to IV and LVEF ≤40%	Ferritin 15-100 ng/mL or between 100-299 ng/mL with a transferrin saturation below 20%	111/114	63 (54-71)	63 (55-70)	67/44	78/36	Polysaccharide iron complex, 150 mg, twice daily	Sugar capsule, 150 mg, twice daily	16 weeks	①③⑤⑥
Wu Y 2013 [41]	NYHA II through IV and LVEF ≤45%	Serum ferritin levels <100 ng/mL	28/28	70 ± 14	70 ± 10	28/8	19/9	Polysaccharide iron complex, 150 mg, once daily	Intravenous sucrose iron	18 weeks	②③④⑤

NYHA: New York Heart Association classification; LVEF: left ventricular ejection fraction; ① NT-proBNP; ② LVEF; ③ 6-minute walk distance; ④ hemoglobin; ⑤ serum ferritin; ⑥ quality of life; ⑦ adverse event.

**Table 2:** Results of evidence quality of outcomes.

Outcomes	Risk of bias	Inconsistency	Indirectness	Imprecisions	Publication bias	Quality result
LVEF	Serious limitation^a^	No serious limitation	No serious limitation	Serious limitation^e^	Not detected	Low
NT-proBNP	Serious limitation^a^	Very serious limitation^d^	No serious limitation	Serious limitation^e^	Not detected	Very low
6MWT	Serious limitation^a^	No serious limitation	No serious limitation	Serious limitation^e^	Not detected	Low
Serum ferritin	Very serious limitation^b^	Very serious limitation^d^	No serious limitation	No serious limitation	Not detected	Very low
Hemoglobin	Very serious limitation^b^	Serious limitation^c^	No serious limitation	Serious limitation^e^	Detected^f^	Very low

LVEF: left ventricular ejection fraction; NT-proBNP: N terminal pro B type natriuretic peptide; 6MWT: 6-minute walk distance; a: most information is from trials at unclear risk of bias, which has serious limitations, that raises some doubt about the results; b: most information is from trials at high risk of bias, which has very serious limitations, that seriously weakens confidence in the results; c: represents heterogeneity (*I*^2^>50%), with treating or interpreting by subgroup analysis or sensitivity analysis, but heterogeneity remained 50%-75%; d: represents heterogeneity (*I*^2^>75%), with treating or interpreting by subgroup analysis or sensitivity analysis, but heterogeneity remained >75%; e: results without clinical significance or small sample sizes (<400); f: publication bias may exist when these small sized studies with all the positive or negative results.

## Data Availability

Data would be available pending the request from corresponding authors.
